# Synergistic antitumor immune response mediated by paclitaxel-conjugated nanohybrid oncolytic adenovirus with dendritic cell therapy

**DOI:** 10.3389/fimmu.2024.1355566

**Published:** 2024-05-21

**Authors:** In-Wook Kim, A-Rum Yoon, JinWoo Hong, Dayananda Kasala, Chae-Ok Yun

**Affiliations:** ^1^ Department of Bioengineering, College of Engineering, Hanyang University, Seoul, Republic of Korea; ^2^ Hanyang Institute of Bioscience and Biotechnology (HY-IBB), Hanyang University, Seoul, Republic of Korea; ^3^ Institute of Nano Science and Technology (INST), Hanyang University, Seoul, Republic of Korea; ^4^ GeneMedicine CO., Ltd., Seoul, Republic of Korea

**Keywords:** DC, oncolytic Ad, nanohybrid, therapeutic vaccine, antitumor immune response, T cells, Treg

## Abstract

Dendritic cell (DC)-based vaccines have emerged as a promising strategy in cancer immunotherapy due to low toxicity. However, the therapeutic efficacy of DC as a monotherapy is insufficient due to highly immunosuppressive tumor environment. To address these limitations of DC as immunotherapeutic agent, we have developed a polymeric nanocomplex incorporating (1) oncolytic adenovirus (oAd) co-expressing interleukin (IL)-12 and granulocyte-macrophage colony-stimulating factor (GM-CSF) and (2) arginine-grafted bioreducible polymer with PEGylated paclitaxel (APP) to restore antitumor immune surveillance function in tumor milieu and potentiate immunostimulatory attributes of DC vaccine. Nanohybrid complex (oAd/APP) in combination with DC (oAd/APP+DC) induced superior expression level of antitumor cytokines (IL-12, GM-CSF, and interferon gamma) than either oAd/APP or DC monotherapy in tumor tissues, thus resulting in superior intratumoral infiltration of both endogenous and exogenous DCs. Furthermore, oAd/APP+DC treatment led superior migration of DC to secondary lymphoid organs, such as draining lymph nodes and spleen, in comparison with either monotherapy. Superior migration profile of DCs in oAd/APP+DC treatment group resulted in more prolific activation of tumor-specific T cells in these lymphoid organs and greater intratumoral infiltration of T cells. Additionally, oAd/APP+DC treatment led to lower subset of tumor infiltrating lymphocytes and splenocytes being immunosuppressive regulatory T cells than any other treatment groups. Collectively, oAd/APP+DC led to superior induction of antitumor immune response and amelioration of immunosuppressive tumor microenvironment to elicit potent tumor growth inhibition than either monotherapy.

## Introduction

Dendritic cells (DC) are potent antigen-presenting cells (APC) that present tumor-associated antigen (TAA) to naïve T cells, thereby inducing an adaptive antitumor immune response by activation and recruitment of type 1 helper (Th1) cells and cytotoxic T lymphocytes (CTLs). DC-based therapeutic vaccines have emerged as a promising strategy in cancer immunotherapy due to low toxicity and immune stimulatory attributes. Despite these promising attributes of DC-based therapeutic vaccines, the efficacy of DC as monotherapeutic was insufficient in clinical trials due to highly immunosuppressive environment of clinical tumors (1, 2). In specific, tumors secrete various immunosuppressive factors, such as vascular endothelial growth factor (VEGF), transforming growth factor (TGF)-β, and interleukin (IL)-10, resulting in formation of immunosuppressive network that attenuates the therapeutic potency of immunotherapeutics. Accumulation of immunosuppressive molecules in tumor milieu impairs the recruitment and maturation of immune effector cells (3). In this regard, an additional therapeutic adjuvant is required to maximize the potency of DC therapeutic vaccines in immunosuppressive tumor microenvironment.

Cytokine therapy is another promising strategy for treatment of cancer (4). However, recombinant cytokine therapy in clinical trials demonstrated low therapeutic efficacy and toxicity, thus requiring alternative delivery route for efficient treatment of cancer. Oncolytic adenovirus (Ad)-mediated expression of cytokine in tumor tissue is a promising approach to address the limitations of cytokine therapy (5–7). In detail, oncolytic Ad-mediated cytokine expression is tumor-specific, as the virus selectively replicates and amplifies transgene in tumor cells, ultimately changing immunosuppressive tumor microenvironment to be more susceptible to antitumor immunity (6, 8, 9). The antitumor immune response mediated by oncolytic Ad can function in a synergistic manner with the innately pro-inflammatory nature of the virus, as even the unarmed virus can promote type I interferon (IFN) response in infected cells by promoting release tumor-associated antigens and danger- or pathogen-associated molecular patterns (10–14).

To date, IL-12 and granulocyte-macrophage colony-stimulating factor (GM-CSF), which promote Th1 immune response (15) and promote maturation of APCs (16), respectively, have been most extensively investigated immune stimulatory therapeutic gene candidates for oncolytic virotherapy across numerous clinical trials with varying degree of success (17, 18). Briefly, GMCSF-expressing oncolytic herpes simplex virus, Imlygic, was the first clinically approved oncolytic virotherapy by both United States and European Union, while several other oncolytic viruses either expressing IL-12 or GMCSF as therapeutic gene have completed or under ongoing investigation in phase II or phase III clinical trials (11, 19–22), demonstrating the promising nature of oncolytic viruses armed with single immune stimulatory transgene. We had previously demonstrated that antitumor efficacy of oncolytic Ad can be further enhanced by coexpressing IL-12 and GM-CSF (oAd) in a single vector, as this virus exerted superior antitumor immune response over the control virus expressing either IL-12 or GMCSF alone (23). Further, the potent antitumor efficacy of oAd was synergistically augmented when combined with adoptively transferred DCs, showing enhanced infiltration and activation of immune effector cells in tumor tissues and potent induction of systemic antitumor immunity (5).

To increase therapeutic potential of Ad, researchers examined the combination of Ad and chemotherapeutic drugs (24, 25). Since combination of Ad and paclitaxel (PTX) enhances Ad’s transduction efficacy in both coxsackie and adenovirus receptor (CAR)-positive and -negative cancer cells while oncolytic Ad chemosensitizes cancer cells to PTX (26), PTX is one of the best chemotherapeutic for combination therapy with Ad. However, PTX, due to its highly hydrophobic nature, has poor solubility and its clinical application is limited by its low water solubility, off-target toxicity, and acquired drug resistance (27, 28). To overcome this limitation of PTX, Nam et al., have developed a PTX-conjugated cationic polymer (APP) by combining arginine-grafted bioreducible polymer (ABP) with PEGylated PTX, which overcome the low solubility and poor penetration of PTX into the tumor tissues (29, 30), resulting in enhanced antitumor efficacy in comparison to PTX (31). We have previously demonstrated that APP-coated p53 variant-expressing oncolytic Ad (oAd-vp53/APP) exerted synergistic antitumor effect against both CAR-positive and -negative breast cancer xenografts via either intratumoral or systemic administration due to enhanced accumulation of both PTX and oncolytic Ad in tumor tissues (32). In our present study, we have utilized APP-complexed oncolytic Ad co-expressing IL-12 and GM-CSF (oAd/APP) in combination with DC to enhance the delivery of PTX and oncolytic Ad to tumor tissue, improve DC function, and subsequently induce potent antitumor effect by conjoining oncolytic Ad, chemotherapeutic, and immunotherapeutic. oAd/APP in combination with DC elicited strong and synergistic antitumor effects via either local or systemic administration, elucidating that oAd/APP can act as a potent adjuvant for optimizing DC vaccination and induction of potent tumor-specific adaptive immunity.

## Materials and methods

### Cell lines and culture

Murine melanoma cell line (B16-F10) and human embryonic kidney cell line transformed with Ad type 5 E1 gene (HEK293) were purchased from the American Type Culture Collection (ATCC, Manassas, VA). Dulbecco’s modified Eagle’s medium (DMEM; Gibco BRL, Grand Island, NY) supplemented with 10% fetal bovine serum (FBS; Gibco BRL), L-glutamine (2 mmol/L), penicillin (100 IU/mL), and streptomycin (50 μg/mL) was used as the culture medium. All cell lines were cultured at 37°C in a humidified atmosphere 5% CO_2_ and 95% air.

### Mice

Six-week-old male C57BL/6 mice were obtained from Orient Bio Inc. (Seongnam, South Korea). Green fluorescent protein (GFP) transgenic mice were purchased from Jackson Laboratories (Bar Harbor, ME). All animal studies were performed according to the institutionally approved protocols of Hanyang University. During the experiments, all mice were kept in a laminar air flow cabinet under specific pathogen free conditions.

### Preparation of oncolytic Ads

The generation and construction of oncolytic Ad coexpressing IL-12 and GM-CSF (oAd) have been described in a previous paper (5). All viruses were propagated in HEK293 cells and purified by CsCl gradient centrifugation (33). oAd was stored at –80°C until use. Numbers of viral particles (VPs) were calculated from optical density measurements at 260 nm (OD_260_), where 1 absorbance (OD_260_ = 1.0) was equivalent to 1.1 × 10^12^ viral particles (VP)/mL.

### Characterization of oAd and oAd/APP

For the physiochemical characterization of oAd/APP complex, 1 × 10^10^ VP of oAd particles and APP solution were gently mixed in phosphate-buffered saline (PBS) by pipetting. The mixtures were allowed to electrostatically interact to form oAd/APP polyplex at room temperature for 30 min, generating oAd/APP complex at an Ad:polymer molar ratio of 1: 1.75 × 10^4^, 8.75 × 10^4^, 3.5 × 10^5^, and 8.75 × 10^5^. The average particle sizes and zeta potentials of oAd and oAd/APP complexes were measured by Zetasizer Nano ZS (Malvern Instrument, Inc., Worcestershire, UK) with a He–Ne laser beam (633 nm, fixed scattering angle of 90°) at 25°C. All other experiments utilized optimal oAd: APP molar ratio of 1:3.5 × 10^5^ as determined in our previous report (32).

### Generation of bone marrow-derived DC

Bone marrow cells were harvested from flushed marrow cavities of femurs and tibias of GFP transgenic mice (Jackson Laboratories) under aseptic conditions. The harvested bone marrow cells were cultured according to a previously reported procedure to isolate GFP-expressing DCs and DCs were subsequently matured by co-incubating with B16-F10 tumor lysates and lipopolysaccharide (5, 9). Immature DCs were prepared in similar manner as mature DCs with only difference being that cells were not exposed to the tumor lysate and lipopolysaccharide.

### Quantification of IL-12, GM-CSF, and IFN-γ expression level

At 48 hr after infection of B16-F10 cells with oAd or oAd/APP at a multiplicity of infection (MOI) of 10, 20, or 50, supernatants were harvested and the level of IL-12 and GM-CSF were determined with conventional IL-12 and GM-CSF ELISA kit (R&D systems, Minneapolis, MN) according to manufacturer’s instruction. To identify level of cytokine expression in tumor tissue, C57BL/6 mice with subcutaneously established B16-F10 melanoma tumor tissues were harvested from mice at 4 days after final treatment with intratumorally administered DCs (1 × 10^6^ cells, 2 × 10^8^ VP of oAd/APP, or combination of oAd/APP+DC, along with PBS group as a negative control. The tissues were homogenized and liquefied in the protease inhibitor cocktail (Sigma-Aldrich, St Louis, MO). IL-12, GM-CSF, and INF-γ level were measured by ELISA kits (R&D systems). To determine serum IL-12 and GM-CSF levels following intratumoral or intravenous oAd/APP administration *in vivo*, mice harboring B16-F10 tumors (mean tumor volume of 300 mm^3^) were treated three times with 1 × 10^10^ VP or 2 × 10^9^ VP of oAd/APP over 3 day-period via intratumoral or intravenous administration, respectively, along with intratumorally administered PBS as negative control group (n=3 per group). The blood samples were obtained from retro-orbital plexus at 3 days after the final treatment, which were centrifuged to obtain the serum. The serums were analyzed by conventional IL-12 and GM-CSF ELISA kit (R&D systems) according to manufacturer’s instructions.

### Established tumor models for *in vivo* antitumor effect

B16-F10 cells (5 × 10^5^) were injected subcutaneously into the right abdomen of 6–7 weeks-old male C57BL/6 mice. When the average tumor volume reached of around 100 mm^3^, animals were sorted into groups with similar mean tumor volumes to begin treatment (designated as day 1 of treatment). Treatment groups included DCs (1 × 10^6^ cells/injection), oAd/APP (2 × 10^8^ or 1 × 10^10^ VP for intratumoral injection, 2 × 10^9^ VP for intravenous injection), or combination of oAd/APP and DC (oAd/APP+DC), along with PBS group as a negative control. Tumor-bearing mice were intratumorally or intravenously injected three times with oAd/APP on day 1, 3, and 5 while mature DC was intratumorally administered three times on days 2, 4, and 6. Tumor growth was monitored day after day using a caliper, and tumor volume was calculated by the following formula: volume = 0.523 × L × W^2^, where L is length and W is width. Animals with tumors that were > 3,000 mm^3^ were euthanized for ethical reasons.

### Histological and immunohistochemical analysis

Tumor tissue were harvested from mice after 9 days of the final injection, frozen in OCT compound (Sakura Finetec, Torrance, CA), and cut into 9-mm cryosections. The cryosections were stained with hematoxylin & eosin (H & E) and then observed by light microscopy. To detect lymphocytes infiltration into tumor tissues, the cryosections were immunostained with purified rat anti-mouse CD4 monoclonal antibody (Ab; BD Biosciences, San Jose, CA) or purified rat anti-mouse CD8 monoclonal Ab as a primary Ab, and then with biotin-conjugated goat anti-rat IgG Ab (BD Biosciences) as a secondary Ab for 1 hr. Subsequently, the sections were incubated with peroxidase-conjugated streptavidin (BD Biosciences). The sections were further incubated with diaminobenzidine (DAB) (DAKO, Copenhagen, Denmark) as the chromogen substrate. All slides were counterstained with Meyer’s hematoxylin (Sigma-Aldrich). To identify GFP-expressing DCs, the cryosections were immunofluorescence stained with hamster anti-mouse CD11c Ab (BD Biosciences), rat anti-mouse CD86 Ab (BD Biosciences), and rabbit anti-human GFP Ab (Millipore, Bedford, MA). Sections were incubated with primary Abs at 4°C overnight, and then incubated Alexa Flour 568-conjugated goat anti-mouse Ab and Alexa 488-conjugated goat anti-rabbit Ab as a secondary Abs for 1 hr. Nuclear staining with 4’,6-diamidino-2-phenylindole (DAPI, Sigma-Aldrich) was also performed. Sections were analyzed by fluorescence microscopy using an IX81-ZDC inverted fluorescence microscope (Olympus Optics, Tokyo, Japan). To detect myeloid-derived suppressor cells (MDSC) infiltration into the tumor tissues, the B16-F10 tumor cryosections were immunostained with PE rat anti-mouse CD11b monoclonal Ab (BD Biosciences) and purified rat anti-mouse Ly-6G/6C (GR-1) Ab as a primary Ab for 2 hr, and then incubated with Alexa Flour 488-conjugated goat anti-rat Ab as a secondary Ab for 1 hr. Nuclear staining with DAPI (Sigma-Aldrich) was also performed. Sections were analyzed by fluorescence microscope (Nikon Ci-L, Japan). Double-stained (PE and 488; red and green) cell counts from four different images for each group were counted with the ImageJ Software (version 1.50b; U.S. National Institutes of Health, Bethesda, MD).

### Fluorescence-activated cell sorting analysis

To investigate the ability of DCs to migrate into regional lymph nodes and spleen *in vivo*, the DCs were stained with surface molecules using immunofluorescence and analyzed by FACS analysis. Mature DCs (1 × 10^6^ cells), oAd/APP (1 × 10^9^ VP) alone, or oAd/APP+DCs were intratumorally injected into established B16-F10 tumors. At 4 days after final injection, the draining lymph nodes (DLNs) and spleen were harvested and stained with FITC-conjugated hamster anti-mouse CD11c Ab (BD Biosciences) or PE-conjugated rat anti-mouse CD86 Ab (BD Biosciences) at 4°C for 45 min. For the assessment of regulatory T (Treg) cell populations by flow cytometry, splenocytes, DLNs, and tumor infiltrating lymphocytes (TIL)s were harvested at 11 days after the initial treatment of B16-F10 tumor-bearing mice with intravenously administered oAd/APP (2 × 10^9^ VP) with or without DC (1 × 10^6^ cells). Cells were pretreated with saturating anti-CD16/CD32 Ab (Biolegend, San Diego, CA) in staining buffer (2% FBS, 0.02% sodium azide in PBS) to block cellular Fc receptors. Cells were stained extracellularly with peridinin chlorophyll protein-Cy5.5-conjugated anti-CD4 Ab (BD Biosciences) and phycoerythrin-conjugated anti-CD25 Ab (eBioscience, San Diego, CA). Cells were then permeabilized with Foxp3 fixation/permeabilization buffer (eBioscience) according to the supplier’s protocol and stained with allophycocyanin-conjugated anti-Foxp3 Ab (eBioscience). To investigate the changes to the immune cell populations of thymus and bone marrow following intratumoral administration of oAd/APP, B16-F10 tumor-bearing mice were intratumorally injected three times with oAd/APP (1 × 10^9^ VP) every other day. Single cells were obtained from thymus and bone marrow harvested at 3 days after the final injection then stained with FITC-conjugated hamster anti-mouse CD3 Ab, peridinin chlorophyll protein-Cy5.5-conjugated anti-CD8 Ab, or APC-Cy7 conjugated anti-CD45 Ab (BD Biosciences) at 4°C for 45 min. All samples were analyzed on a BD Biosciences BD-LSR II Analytic Flow Cytometer, using FACSDiva software (BD Biosciences).

### Statistical analysis

Data are expressed as mean ± standard deviation (SD). Statistical significance was determined by two-tailed Student t-test (SPSS 13.0 software; SPSS, Chicago, IL, USA). *P*-values < 0.05 were considered statistically significant (*P < 0.05, **P < 0.01, ***P < 0.001).

## Results

### Characterization of Ad/APP complex-mediated therapeutic gene expression

**Figure 1:**
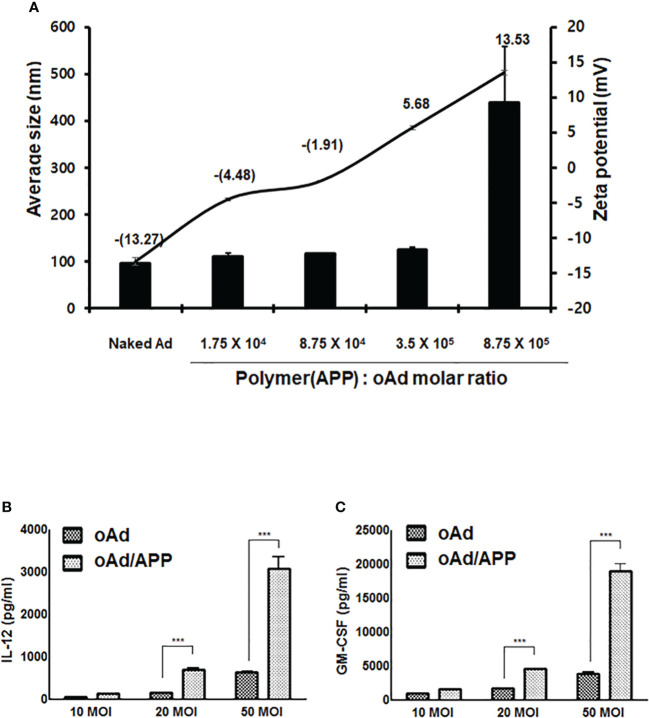
Physicochemical characterization of oAd/APP complex and complex-mediated therapeutic gene expression. The average size (nm) and zeta potential value of the oAd/APP complex were measured at various molar ratios indicated **(A)**. The size and charge determination are the mean ± SD of three independent experiments. The expression level of IL-12 **(B)** and GM-CSF **(C)** were assessed in B16-F10 cells. Cells were infected with oAd or oAd/APP and the supernatant were collected at 48 hr after infection. The level of IL-12 and GM-CSF was quantified by conventional ELISA. Data represent the mean ± SD of triplicates and similar results were obtained from at least three separate experiments. MOI, multiplicity of infection. ***P < 0.001.

We have previously demonstrated that Ad can be efficiently encapsulated by PTX-conjugated polymer micelle, APP, by electrostatic interaction between negatively charged Ad surface and cationic polymer, generating a cationic polyplex that could co-deliver oncolytic Ad and PTX into tumor tissues (32). A new batch of APP polymer was synthesized and different oncolytic Ad was utilized in present report, thus we assessed whether new oAd/APP complex retained similar physiochemical attributes as those generated in our previous report utilizing oAd-vp53/APP by measuring average size and zeta potential. As shown in **Figure 1A**, the average size and zeta potential of naked oAd were 96.9 ± 5.1 nm and -13.3 ± 2.1 mV, respectively. Complexation of oAd with APP at Ad:polymer molar ratios ranging from 1:1.75 × 10^4^ to 3.5 × 10^5^ led to polymer concentration-dependent increase in average size and zeta potential (at 3.5 × 10^5^ molar ratio; 125.8 ± 3.1 nm and 5.7 ± 0.3 mV). The steep increase in complex size and charge at the molar ratio of 8.75 × 10^5^ was due to aggregation, which is in line with our previous report where aggregation was observed at the molar ratio (32). Based on these current and past results, APP:oAd molar ratio of 3.5 × 10^5^ was chosen as optimal molar ratio and used in all of the subsequent experiments.

To evaluate whether oAd/APP complex can effectively infect our target B16-F10 murine melanoma cells to induce expression of therapeutic genes, B16-F10 cells were infected with naked oAd or oAd/APP at an MOI of 10, 20, or 50 and the expression level of IL-12 or GM-CSF was measured by ELISA. As shown in **Figures 1B, C**, both naked oAd and oAd/APP showed dose-dependent increase in expression levels of IL-12 and GM-CSF. Importantly, the expression level of IL-12 and GM-CSF expression induced by oAd/APP was significantly higher than those of naked oAd (at an MOI 20 and 50; *P <* 0.001) suggesting that APP-mediated delivery of oAd can enhance the therapeutic gene expression in murine melanoma cells.

### Antitumor effect of oAd/APP in combination with dendritic cells

**Figure 2:**
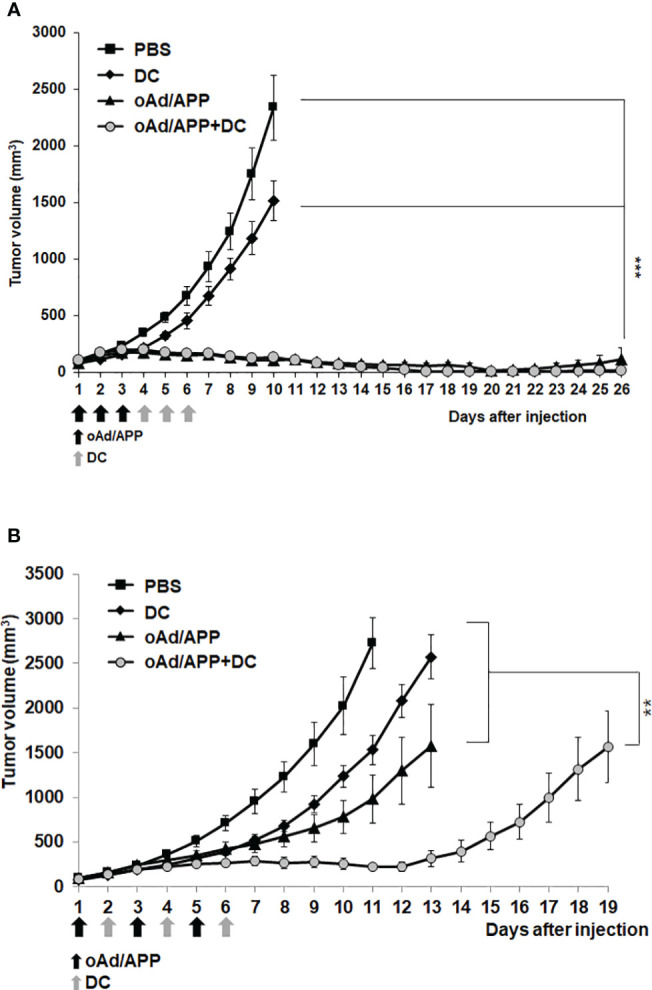
Potent antitumor effect of oAd/APP in combination with DCs. Pre-established B16-F10 tumors were injected with phosphate-buffered saline (PBS), DCs, oAd/APP complex (1 × 10^10^ **(A)** or 2 × 10^8^ **(B)** VP, respectively), or oAd/APP plus DCs (1 × 10^6^ cells). Tumor growth was monitored every day, data points represent the mean ± SE of the tumor size in each group (n = 6). ***P < 0.001 oAd/APP versus PBS or DC, ***P < 0.001 oAd/APP+DC versus PBS or DC, not significant (NS) for oAd/APP versus oAd/APP+DC. **(B)** **P < 0.01, oAd/APP+DC versus oAd/APP. (**P < 0.01, ***P < 0,001).

To evaluate the therapeutic efficacy of oAd/APP in combination with DCs *in vivo*, C57BL/6 mice were subcutaneously inoculated with B16-F10 melanoma cells. When the average tumor volume reached 100 mm^3^, mice were intratumorally treated with PBS, DC, oAd/APP, or oAd/APP+DC (designated as Day 1). Based on our previous experience combining oAd with DC (5, 34), oAd/APP was administered every day in a consecutive manner (Day 1–3) followed by three consecutive dosing of DC (day 4–6). As shown in **Figure 2A**, mice treated with PBS control showed rapid and aggressive tumor growth and tumor volume exceeded 2,000 mm^3^ at 10 days following initial treatment. In marked contrast, mice treated with either oAd/APP or oAd/APP+DC combination showed significant inhibition of tumor growth in comparison to those treated with PBS or DC alone, resulting in complete tumor regression at day 27 following initial treatment (4/6 in oAd/APP and 6/6 in oAd/APP+DC group). Furthermore, oAd/APP+DC induced complete tumor regression at earlier time points (day 14, 16, 17, and 19) than oAd/APP (day 19 and 20), suggesting that combination of oAd and DC may induce more rapid and efficient induction of antitumor immune response. At earlier time point (20 days post initial treatment), there were 4 mice without any observable tumors in oAd/APP-treated group, however, only 2 of these mice remained tumor free at 26 days post injection due to tumor regrowth. In marked contrast, 100% of oAd/APP+DC- treated mice remained free of tumor at 26 days post injection, suggesting that combination of oAd/APP with DC vaccine induced durable remission.

As it was difficult to distinguish the difference in tumor growth inhibiting effect of oAd/APP and oAd/APP+DC at the viral dose (1 × 10^10^ VP) utilized in **Figure 2A**, the therapeutic efficacy of the combination therapy utilizing lower viral doses (5 × 10^7^ or 2 × 10^8^ VP) under the same treatment schedule were investigated. Unexpectedly, the combination therapy of oAd/APP at 5 × 10^7^ or 2 × 10^8^ VP and DC did not elicit superior antitumor efficacy over respective dose of oAd/APP monotherapy (**Supplementary Figure S1**), suggesting that the current treatment schedule based on our previous studies (5, 34) was suboptimal for present study utilizing APP as nanocarrier for oncolytic Ad. Thus, alternative dosing regimen utilizing 2 × 10^8^ VP of oAd/APP was investigated. As shown in **Figure 2B**, alternating oAd/APP (Day 1, 3, and 5) and DC (Day 2, 4, and 6) treatment led to combination therapy (oAd/APP+DC group) exerting significantly more potent antitumor efficacy than either DC or oAd/APP monotherapy (*P* < 0.01), demonstrating that combination of both therapeutics under optimal treatment schedule can induce beneficial tumor growth inhibiting effect.

### Intratumoral expression of IL-12, GM-CSF and INF-γ

**Figure 3:**
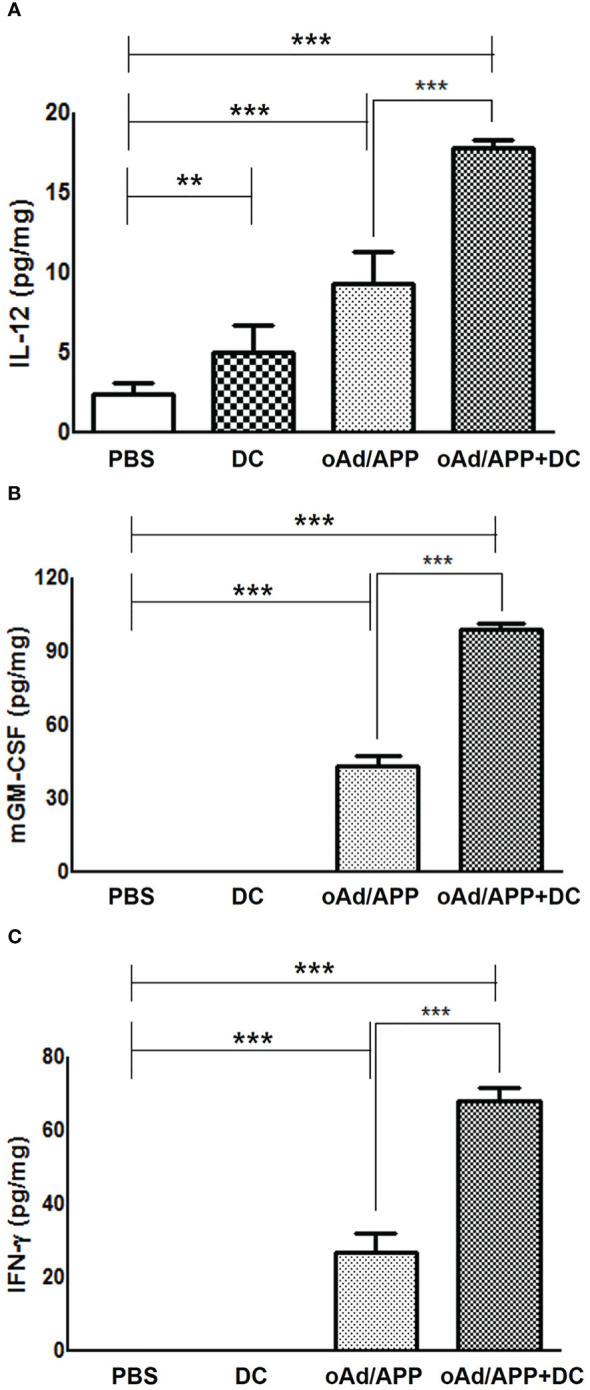
Intratumoral expression of IL-12, GM-CSF and INF-γ. Subcutaneously established B16-F10 melanoma tumor tissues were harvest at 4 days after the final intratumoral treatment with 2 × 10^8^ VP of oAd/APP and/or 1 × 10^6^ DCs. ELISA was performed to evaluate the expression level of **(A)** IL-12, **(B)** GM-CSF, **(C)** IFN-γ in tumor. Experiment were carried out in triplicates (n =3 mice per group). Each data point indicated mean ± SD. (**P < 0.01, ***P < 0,001).

To further elucidate the mechanism behind the antitumor effect of each treatment, intratumoral expression level of IL-12, GM-CSF, and IFN-γ were examine by ELISA from tumor homogenate collected at 4 days post last treatment. As shown in **Figure 3**, all cytokines (IL-12, GM-CSF, and IFN-γ) were either not detected or detected at a low level in tumors treated with PBS or DC. In marked contrast, both oAd/APP and oAd/APP+DC treatment resulted in significantly higher expression level of cytokines. Importantly, tumor treated with oAd/APP+DC group showed significantly higher concentration of all cytokines than oAd/APP group (*P* < 0.001), demonstrating that both therapeutics can conjointly enhance expression of antitumor cytokines in immunosuppressive tumor.

### Immune cell infiltration in tumor tissues treated with combination of oAd/APP and DCs

**Figure 4:**
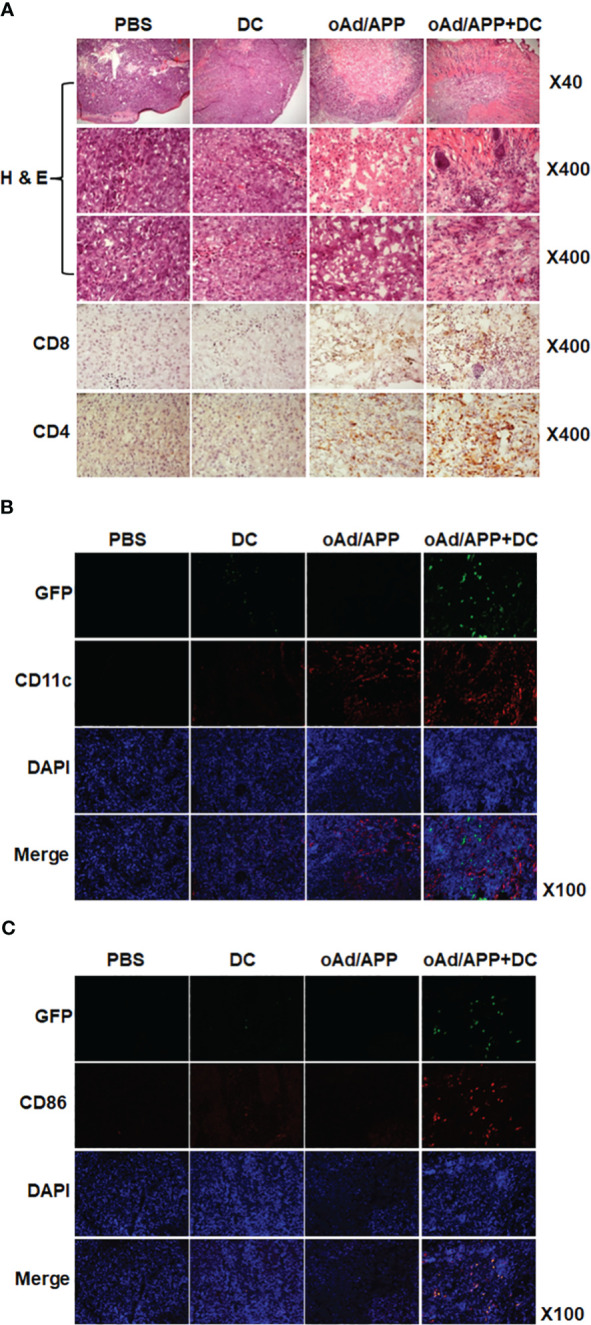
Immune cell infiltration into tumor tissues. Tumor tissues were harvested from mice at 15 days after the initial treatment for histological analysis **(A)** frozen section of the tumor was stained with hematoxylin and eosin (H & E). Frozen section of tumor tissue was stained with anti-CD4 and anti-CD8 antibodies. **(B)** Frozen sections were stained with anti-CD11c and anti-GFP antibodies. **(C)** Frozen sections were stained with anti-CD86 and anti-GFP antibodies. Original magnification: ×40, ×100, or ×400.

To further assess the antitumor immune response mediated by each treatment, histology and immune cell infiltration in the tumor tissues were examined by immunohistochemical staining of tumors harvested on 15 days after the initial treatment. As shown in **Figure 4A**, H & E staining revealed extensive accumulation of tumor cells in large areas of the PBS- or DC-treated tumor tissues. In contrast, a large portion of oAd/APP-treated tissues was necrotic, reaffirming the potent antitumor efficacy of oAd/APP. Interestingly, oAd/APP+DC-treated tumor tissues showed markedly lower tumor cell population than any other treatment groups and extensive accumulation of normal cells were observed, suggesting that rapid induction of potent antitumor effect may contribute to proliferation of normal cells and expedite tissue recovery.

The tumor tissues were immunohistochemically stained with CD4-, CD8-, CD11c-, CD86- and GFP-specific Ab to assess immune cells infiltration. Both oAd/APP and oAd/APP+DC treatment induced higher level of CD4- or CD8-positive T cell infiltration into tumor tissues than PBS or DC treatment, with oAd/APP+DC leading to more robust infiltration than oAd/APP monotherapy. Similar results were obtained with intratumoral infiltration of DCs (CD11c^+^) where both oAd/APP and oAd/APP+DC treatment led to markedly higher infiltration of DCs than those treated with PBS or DC (**Figure 4B**). Importantly, oAd/APP+DC showing markedly higher quantity of both endogenous and exogenous DCs (GFP^-^CD11c^+^ and GFP^+^CD11c^+^) than oAd/APP monotherapy. Of note, both endogenous and exogenous mature DCs (GFP^-^CD86^+^ and GFP^+^CD86^+^, respectively) were only detected in oAd/APP+DC combination therapy group (**Figure 4C**).

Next, we investigated the effect of each treatment on myeloid-derived suppressor cell (MDSC) population within the tumor microenvironment, as high level of MDSC can abrogate the therapeutic benefit of DC vaccine and recombinant GM-CSF therapy has been reported to promote MDSC accumulation in the tumor tissues (35–39). As shown in **Supplementary Figure S2**, the tumor tissues treated with PBS demonstrated highest number of CD11b^+^GR-1^+^ MDSC accumulation followed by oAd/APP monotherapy group, suggesting that GM-CSF as therapeutic transgene in our system did not increase MDSC population within the tumor. In sharp contrast, both DC and oAd/APP+DC treatment induced similarly potent reduction of MDSC population within the tumor tissues, which was in agreement with previous reports demonstrating that tumor lysate pulsed mature DC vaccine therapy can attenuate MDSC accumulation in the tumor tissues (40, 41). These findings demonstrated that high level of GM-CSF expression induced by oAd/APP did not promote recruitment of immunosuppressive MDSCs, and its combination with DCs can be an effective approach to attenuate MDSC accumulation in the tumor.

Together, these results suggest that oAd/APP can enhance retainment of exogenously administered DCs, activation of endogenous DCs, and infiltration of immune effector cells (T cells and DCs) to tumor tissues.

### DC migration to draining lymph nodes following treatment with combination of oAd/APP and DCs

**Figure 5:**
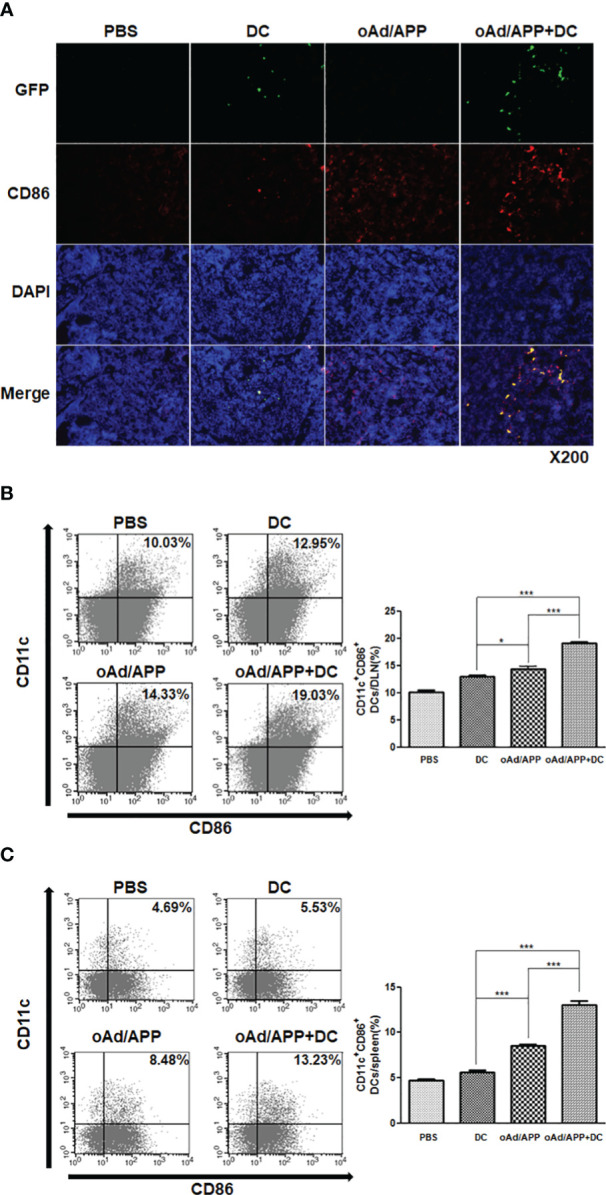
DC migration activity after treatment with combination of oAd/APP and DCs. Four days after the final treatment, single cells were collected from draining lymph nodes (DLN)s and spleen. **(A)** Frozen section of the DLN was stained anti-CD86 and anti-GFP antibodies. Original magnification: ×200. The migration DC was quantified by FACS. The population of CD11c^+^CD86^+^ cells in draining lymph nodes **(B)** and spleen **(C)** from mice treated with phosphate-buffered saline (PBS), DC, oAd/APP, or oAd/APP+DCs. *P < 0.05, ***P < 0.001.

To assess whether each treatment can promote DC migration to DLNs to promote adaptive immune response, tumors were intratumorally injected with PBS, DC, oAd/APP, or oAd/APP+DC. Four days after the final injection, DLNs were harvested and stained with GFP, CD11c, and CD86-specific Ab. As shown in **Figure 5A**, both oAd/APP and oAd/APP+DC treatment led to high level of migration for endogenous mature DCs (GFP^-^CD86^+^) than either PBS or DC treatment, suggesting that cytokine expression mediated by oAd/APP could promote DC maturation and migration to DLNs. Importantly, oAd/APP+DC treatment led to markedly higher number of both endogenous and exogenous mature DCs migrating to DLNs than any other treatment group, demonstrating that combination therapy can promote migration of both DC vaccine and endogenous DCs to DLNs and mount an effective antitumor immune response.

To further assess whether combination therapy increased the accumulation of activated DC population in secondary lymphoid organs, we examined the CD11c^+^CD86^+^ cell population in DLN and spleen by flow cytometry. Similar to our results from **Figure 5A**, both oAd/APP and oAd/APP+DC treatment led to markedly higher number of mature DCs (CD11c^+^CD86^+^) being detected in DLNs than either PBS or DC treatment (*P* < 0.05 or 0.001), respectively. As expected, oAd/APP+DC treatment led to significantly higher quantity of mature DCs being detected in DLNs than those treated with oAd/APP monotherapy (*P* < 0.001) (**Figure 5B**). Similar trends were observed in the spleen, in which oAd/APP+DC treatment led to significantly higher accumulation of mature DCs (CD11c^+^CD86^+^) than any other treatment group (**Figure 5C**; *P* < 0.001). These results suggest that combination therapy promoted migration of DC to secondary lymphoid organs.

### Antitumor effect of systemically injected oAd/APP in combination with dendritic cells

**Figure 6:**
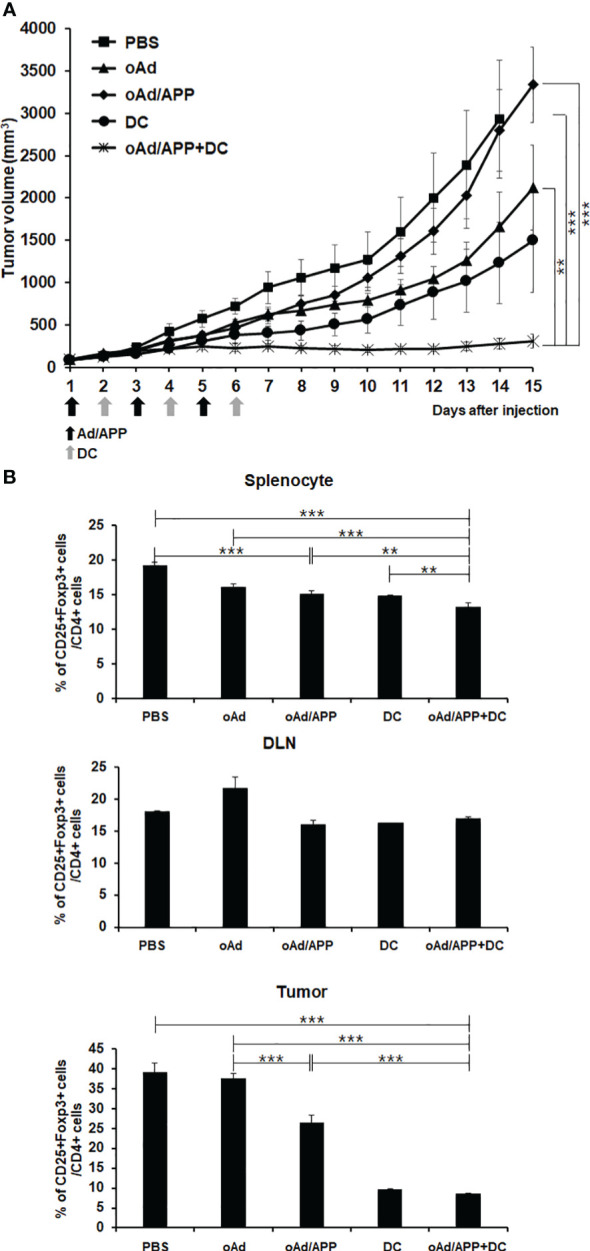
Antitumor effect and Treg population after systemic administration of Ad/APP in combination with dendritic cells. **(A)** Mice harboring established B16-F10 tumors were intravenously injected PBS, DCs (1 × 10^6^ cells), oAd/APP complex (2 × 10^9^ VP), or oAd/APP+DCs. Tumor growth was monitored every day, data points represent the mean ± SE of the tumor size in each group (n = 6). At Day 15, ***P < 0.001; oAd/APP+DC versus PBS or oAd/APP, **P < 0.01; oAd/APP+DC versus DC, and non-significant (NS); oAd/APP versus DC. **(B)** Treg cell population was analyzed that splenocyte, DLNs and tumor-infiltrating lymphocyte (TIL) were harvested at 11 days following final viral injection of B16-F10 tumor-bearing mice by oAd/APP (2 × 10^9^ VP) intravenous injection and oAd/APP (2 × 10^9^ VP)+DC (1 × 10^6^ cells; intratumoral injection) by analyzing the CD25, Foxp3, and CD4 expression level with flow cytometry. Experiment were carried out triplicate (n =3 mice per group). Each data point indicated mean ± SD. (**P < 0.01, ***P < 0,001).

To date, locoregional injection of the oncolytic virus remains the preferred administration route in clinical trials due to potential safety concerns, like immune-related adverse events and off-target toxicity, and inadequate therapeutic benefit by systemic administration (42, 43). Effective systemic application of oncolytic viruses remain a major goal to maximize the therapeutic potential of the virus against inaccessible tumors or distant metastases. As we had previously demonstrated that systemic administration of oAd/APP induced tumor growth inhibition in a safe manner (32), we sought to investigate whether intravenously administered oAd/APP could exert synergistic antitumor effect in combination with DCs. B16-F10 tumor-bearing mice treated with PBS control showed rapid and aggressive tumor growth, and tumor volume exceeded 3,000 mm^3^ at 15 days following initial treatment (**Figure 6A**). oAd treatment alone also led to aggressive tumor growth and rapidly formed large tumors (over 2,500 mm^3^) at day 13. In contrast, mice treated with oAd/APP or DCs alone showed significant inhibition of tumor growth. Moreover, mice treated with oAd/APP+DC showed synergistic antitumor effects that more inhibition of tumor growth than oAd/APP or DCs alone groups. On day 15, the mean tumor volumes in mice treated with oAd alone, oAd/APP, DC alone, or oAd/APP+DC groups were 3,335 ± 473, 2,121 ± 377, 1,494 ± 362, and 311 ± 29 mm^3^, respectively. Tumor growth inhibition was statistically significant in mice treated with oAd/APP+DC as compared with individual treatment groups (*P* < 0.05 versus DCs, *P* < 0.01 versus oAd/APP, *P* < 0.05 versus oAd or PBS), respectively.

To assess whether effective activation and infiltration of immune cells were due to amelioration of immunosuppressive tumor microenvironment, the changes to CD4^+^CD25^+^Foxp3^+^ immunosuppressive Treg cell population among splenocytes, DLN cells, and TILs were analyzed by flow cytometry. Mice treated with oAd/APP+DC showed significantly attenuated triple positive Treg populations in spleen, DLN, and TILs compared with PBS-, DC- and oAd/APP-treated mice (**Figure 6B**). Collectively, these results demonstrate that accumulation of immunosuppressive Treg cells in tumor microenvironments and lymphoid organs was effectively attenuated by oAd/APP+DC, leading to potent induction of antitumor immune response.

## Discussion

We have previously demonstrated that Ad can be efficiently encapsulated by PTX-conjugated polymer micelle (APP), which showed a much higher transduction efficiency in both CAR-positive and -negative cancer cells, ultimately eliciting synergistic antitumor effect (32). oAd/APP complex retained similar physiochemical attributes (average size and surface charge) as those generated in our previous report and showed greatly enhanced expression level of cytokines compared to oAd alone (**Figure 1**). This is in line with several other reports demonstrating that combination of PTX and oncolytic Ad can enhance Ad-mediated transgene expression by mechanisms, such as increased expression of cellular uptake receptors-targeted by Ad (44), induction of G2/M cell cycle arrest (45), or induction of aberrant mitosis (46).

DCs, which act as professional antigen presenting cells, are crucial for activation of antitumor immune responses (47). However, DC vaccines in clinical trial as monotherapy have shown limited efficacy due to immunosuppressive tumor microenvironment preventing infiltration and activation of immune effector cells (48). In this regard, oncolytic Ad, which is not toxic to monocyte-derived immature DCs, is a particularly promising candidate for boosting DC vaccine as several reports have demonstrated antitumor immune activation by virus-mediated oncolysis and subsequent generation of tumor-associated antigens (49). This immune stimulatory attributes of oncolytic Ad can be further augmented by arming these viruses with immune stimulatory cytokines, which led to amelioration of the immunosuppressive tumor microenvironment and DC maturation (5, 9, 33). Importantly, cytokine-expressing oncolytic Ad can act as a potent immune adjuvant to promote Th1 antitumor immune response (50, 51) and overcome the obstacles of DC vaccination (52). oAd mediated expression of IL-12 enhances Th1 immunity and activate immune cells (e.g., CTLs and natural killer (NK) cells) and these cells can promote DC maturation and DC-mediated IL-12 secretion, resulting in a positive feedback loop that potentiates immune cells to elicit a potent antitumor immune response (53). In line with these findings, oAd/APP in combination with DC led to higher expression level of antitumor cytokines, such as IL-12, GM-CSF, and IFN-γ, in tumor tissues (**Figure 3**), suggesting that DCs stimulated with IL-12 can release IFN-γ and that both immunotherapeutics can synergistically induce cytokine expression (53, 54). Neither intratumoral nor intravenous oAd/APP administration induced upregulation of pro-inflammatory cytokines (IL-12 and GM-CSF) in the serum (**Supplementary Figure S3**), thus minimizing the safety concerns associated with systemic administration of recombinant IL-12. One potential Importantly, a strong positive correlation between antitumor cytokine expression levels and intratumoral infiltration of immune cells (immature and mature DCs as well as CD4^+^ or CD8^+^ T cells) was observed where oAd/APP+DC treatment resulted in highest level of infiltration or retainment of these immune cells (**Figures 3**–**5**), further supporting the notion that inactivation of immune cells by immunosuppressive tumor milieu could be overcome by overexpression of antitumor cytokines. Notably, treatment with oAd/APP+DC led to significantly improved retainment of intratumorally administered exogenous DCs compared to DC alone (**Figures 4B, C**), suggesting that combination therapy attenuates immunosuppression-mediated inactivation and clearance of DCs from tumor tissues.

One of the unexpected finding in this study was that alternating oAd/APP and DC treatments over the course of 6-day interval was necessary for the combination therapy to exert beneficial antitumor effect over respective monotherapy (**Figure 2A**), whereas sequential administration of oAd/APP (day 1–3; one injection/day) followed by DC (day 4–6; one injection/day) failed to exert superior antitumor efficacy over oAd/APP monotherapy). Our previous works utilizing the same oAd (oAd co-expressing IL-12 and GMCSF) in combination with DC revealed that sequential administration of oAd/APP followed by DC treatments was both experimentally and mathematically shown to be one of the optimal arrangements to exert beneficial therapeutic effect (5, 34), suggesting that APP-mediated delivery of oAd may exert profoundly different biological effect over the naked oAd. PTX moiety in APP molecule may contribute to this differential effect, since there are few studies demonstrating that PTX may inhibit pro-inflammatory cytokine (IL-12 and IFN-γ) secretion or deplete DC in tumor tissues (55, 56). Sequential dosing may promote higher level of PTX accumulation (**Figure 2A**; **Supplementary Figure S1**) in the tumor microenvironment compared to alternating dosing regimen where oAd/APP is administered every other day (**Figure 2B**). Still, no definitive conclusion can be made, as several publications demonstrated that PTX can promote maturation and activation DCs (57, 58) and this aspect of oAd/APP warrant further investigation in the future.

Migration of mature DCs to regional lymph nodes is an essential procedure for the induction of tumor-specific immune response by activation and maturation of T cells (59, 60). An increased retainment and infiltration of both exogenous and endogenous DCs via elevated expression of antitumor cytokines was also associated with augmented DC migration toward the DLNs and spleen following oAd/APP+DC treatment (**Figures 5A-C**). This is in line with previous literature demonstrating that increase in expression level of Th1 cytokines and co-stimulatory molecule promotes DC migration to lymphoid organs (61, 62). Additionally, we observed that locally administered oAd/APP elevated CD8^+^ T cell frequency in other distant immune niche, like bone marrow and thymus, compared with PBS control group (**Supplementary Figure S4**), suggesting that activation and maturation of DCs in the lymphoid organs may instigate systemic CD8^+^ T cell immune response.

Systemic administration is paramount in cancer therapy, facilitating the delivery of therapeutic agents across the entire circulatory system. This approach ensures widespread distribution, increasing the probability of reaching metastatic sites and thereby enhancing the efficacy of the treatment against disseminated cancer cells. Intravenous injection of oAd/APP+DC also led to induction of potent antitumor effect (**Figure 6A**) induced an immune response that reduced a number of immune-suppressive factors to circumvent the immunosuppressive microenvironment (**Figure 6B**). These results indicate that injection of the cytokine-expressing oAd/APP+DC can attenuate immunosuppressive Treg cells to enhance the antitumor immune response mediated by DC vaccination. This claim is supported by several other reports demonstrating that increased frequency of Treg cells occurs in patients with malignant tumors and is closely correlated to the poor survival of cancer patients (63, 64). This is due to Treg cells being dominantly responsible for the immunosuppression and impaired immune responses in the cancer-bearing hosts (65). The attenuation in Treg population from TIL, splenocytes, and DLN following oAd/APP+DC treatment was likely due to high intratumoral expression level of IL-12 (**Figures 3A**, **6B**), as localized overexpression of IL-12 in tumor has been reported to reverse the immunosuppression by inducing apoptosis of Treg cells (66). Our findings demonstrated that diverse immune cell subsets (MDSCs, Tregs, CD4^+^ or CD8^+^ T cells, and DCs) across tumor tissues and lymphoid organs were differentially regulated by oAd/APP+DC combination therapy that were suggestive of systemic antitumor immune response. Still, the complex nature (e.g., multiple anticancer modalities) of the system and potentially conflicting functions of some therapeutic components (e.g., GM-CSF transgene) necessitate more comprehensive immune profiling of the combined regimen in the future to ascertain how this system function in different tumor types.

Taken together, this study demonstrates that oAd/APP nanohybrid complex can act as a potent adjuvant for optimizing DC vaccination. The therapeutic benefit of the combination therapy is achieved via enhanced expression of antitumor cytokines, reduction of immunosuppressive cell population in tumor tissues and lymphoid organs, and augmentation of DC activity, which translated to induction of potent antitumor immune response.

## Conclusion

This research aimed to maximize the strengths and overcome the shortcomings of each treatment by fusing three therapeutic platforms: oAd-mediated cancer gene therapy, nanomaterial-based drug delivery system, and dendritic cell therapy-mediated immunotherapy. oAd/APP complex induced high level of pro-inflammatory cytokine expression *in vitro*. Both intratumorally and intravenously administered oAd/APP exerted beneficial tumor growth inhibiting effect in combination with DC vaccination via induction of potent antitumor immune response. Although this study demonstrated that oAd/APP complex combined with DC therapy exhibited a strong antitumor effect under alternating dosing regimen, the beneficial effect of the combination therapy was negated when all doses of oAd/APP were administered prior to DC administration, suggesting that there may be a potentially antagonistic role of APP to DC vaccination therapy that warrants more in-depth investigation in the future. Additionally, the therapeutic efficacy of systemically administered oAd/APP was markedly lower than those achieved via intratumoral optimization, suggesting that further optimization in tumor-targeted delivery efficiency of the APP will be necessary to exert sufficient antitumor effect in a complex clinical tumor microenvironment.

## Data availability statement

The original contributions presented in the study are included in the article/**Supplementary Material**, further inquiries can be directed to the corresponding author.

## Ethics statement

Animal studies were conducted according to the institutional guidelines established by the Hanyang University Institutional Animal Care and Use Committee. The study was conducted in accordance with the local legislation and institutional requirements.

## Author contributions

I-WK: Writing – original draft, Writing – review & editing, Conceptualization, Data curation, Formal analysis, Investigation, Methodology, Validation. A-RY: Writing – original draft, Writing – review & editing, Conceptualization, Data curation, Formal analysis, Funding acquisition, Investigation, Methodology, Project administration. JH: Writing – review & editing, Data curation, Methodology, Supervision, Validation. DK: Formal analysis, Investigation, Methodology, Writing – review & editing. C-OY: Conceptualization, Funding acquisition, Project administration, Resources, Supervision, Writing – original draft, Writing – review & editing.

## Supplementary material

Supplementary Figure 1 Potent antitumor effect of oAd/APP in combination with DCs. Pre-established B16-F10 tumors were injected with phosphate-buffered saline (PBS), 1 × 10^6^ DCs, oAd/APP complex (5 × 10^7^ **(A)** or 2 × 10^8^ **(B)** VP, respectively), or oAd/APP plus DCs. Tumor growth was monitored every day, data points represent the mean ± SE of the tumor size in each group (n = 3 or 4).

Supplementary Figure 2 Myeloid-derived suppressor cells (MDSC) population in tumor tissues. B16-F10 tumor tissues were harvested from mice at 15 days after the initial treatment for histological analysis frozen section of the tumor was stained with anti-CD11b (red) and anti-Gr1(Ly-6G/Ly-6C; green) antibodies. Original magnification: ×100. The number of Cd11b^+^GR-1^+^ cells in each group were semi-quantitatively analyzed using ImageJ software. Each data point indicated mean ± SD. [**P* < 0.05, and non-significant (NS)].

Supplementary Figure 3 Serum level of IL-12 and GM-CSF. Serum samples were harvested from B16-F10 tumor-bearing mouse at 3 days after the third administration of oAd/APP (intratumoral injection = 1 × 10^10^ VP; intravenous injection = 2 × 10^9^ VP per injection). PBS was administered intratumorally as negative control. ELISA was performed to evaluate the serum level of (**A**) IL-12 or (**B**) GM-CSF (n = 3 mice per group). Each data point indicated mean ± SD. Non-significant (NS).

Supplementary Figure 4 Changes in immune cell population within thymus and bone marrow after intratumoral administration of oAd/APP in B16-F10 tumor bearing mice. Thymus **(A, B)** or bone marrow **(C)** was harvested at 3 days after the third administration of oAd/APP complex (1 × 10^9^ VP) into B16-F10 tumor-bearing mice CD3 or CD8 expression levels were analyzed by flow cytometry (n =3 mice per group). Each data point indicated mean ± SD. (***P < 0.01*, *****P* < 0.0001, and non-significant (NS)).
