# Supplementary figures and images for: Synergistic antitumor immune response mediated by paclitaxel-conjugated nanohybrid oncolytic adenovirus with dendritic cell therapy
Source: Front Immunol. 2024 May 21;15:1355566. doi: 10.3389/fimmu.2024.1355566 (PMC11148213; doi:10.3389/fimmu.2024.1355566)

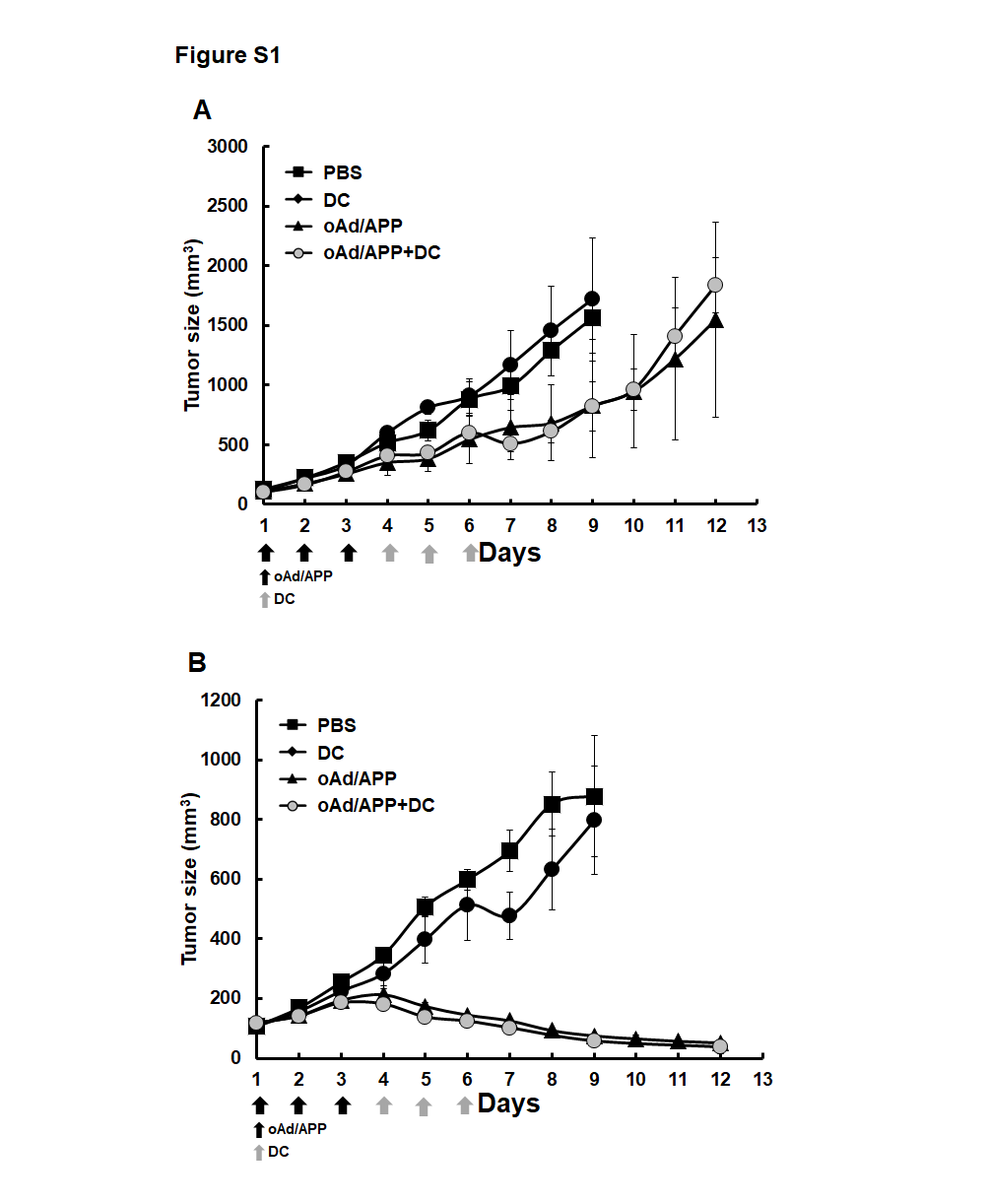

Supplement: Supplementary Figure 1 — Potent antitumor effect of oAd/APP in combination with DCs. Pre-established B16-F10 tumors were injected with phosphate-buffered saline (PBS), 1 × 10^6^ DCs, oAd/APP complex (5 × 10^7^ (A) or 2 × 10^8^ (B) VP, respectively), or oAd/APP plus DCs. Tumor growth was monitored every day, data points represent the mean ± SE of the tumor size in each group (n = 3 or 4). [file Image_1.tif]

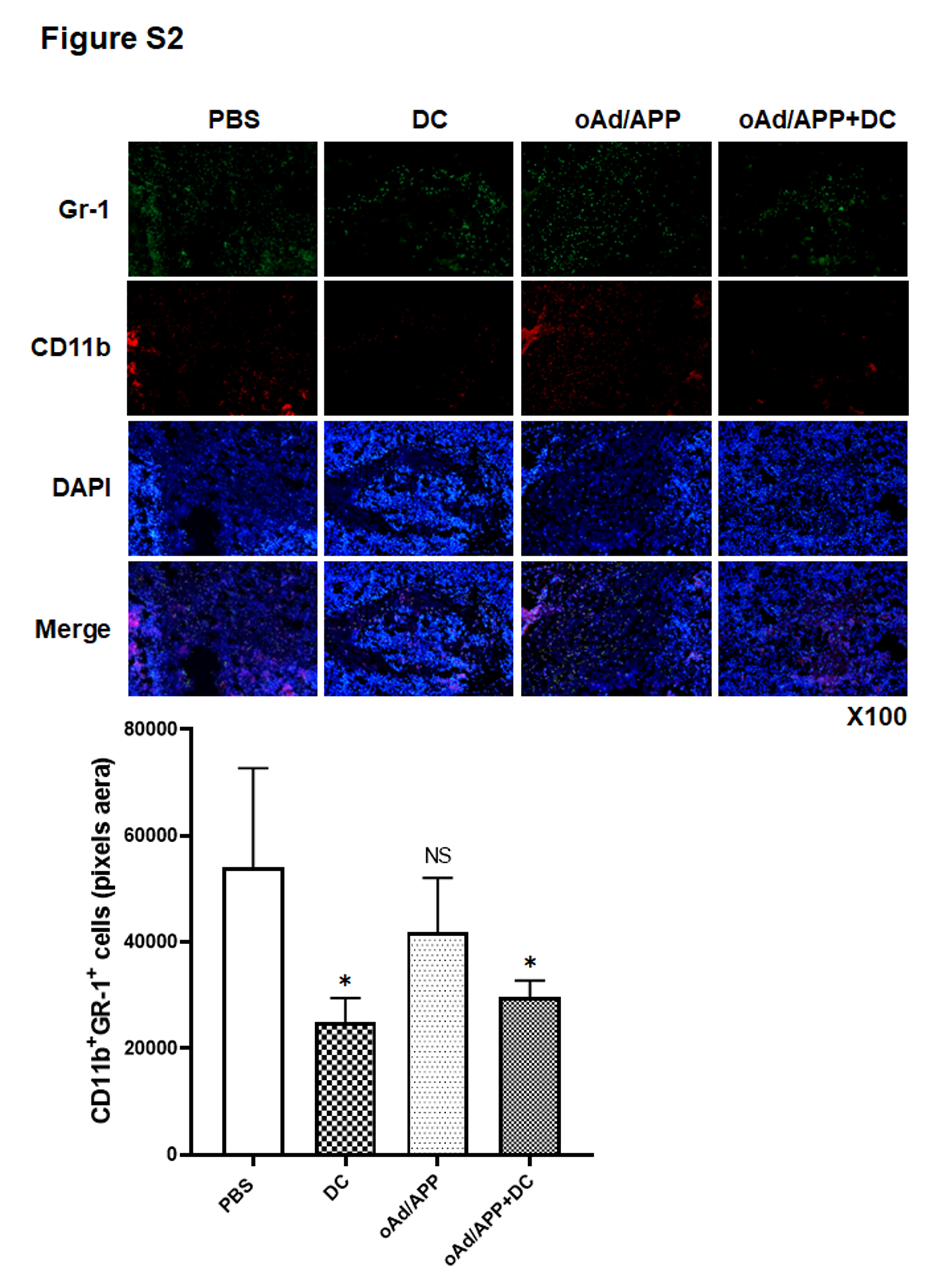

Supplement: Supplementary Figure 2 — Myeloid-derived suppressor cells (MDSC) population in tumor tissues. B16-F10 tumor tissues were harvested from mice at 15 days after the initial treatment for histological analysis frozen section of the tumor was stained with anti-CD11b (red) and anti-Gr1(Ly-6G/Ly-6C; green) antibodies. Original magnification: ×100. The number of Cd11b+GR-1+ cells in each group were semi-quantitatively analyzed using ImageJ software. Each data point indicated mean ± SD. [*P < 0.05, and non-significant (NS)]. [file Image_2.tif]

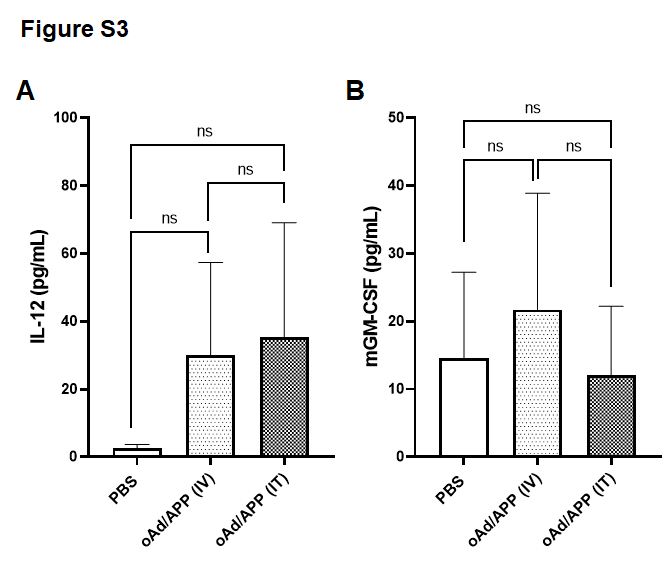

Supplement: Supplementary Figure 3 — Serum level of IL-12 and GM-CSF. Serum samples were harvested from B16-F10 tumor-bearing mouse at 3 days after the third administration of oAd/APP (intratumoral injection = 1 × 10^10^ VP; intravenous injection = 2 × 10^9^ VP per injection). PBS was administered intratumorally as negative control. ELISA was performed to evaluate the serum level of (A) IL-12 or (B) GM-CSF (n = 3 mice per group). Each data point indicated mean ± SD. Non-significant (NS). [file Image_3.tif]

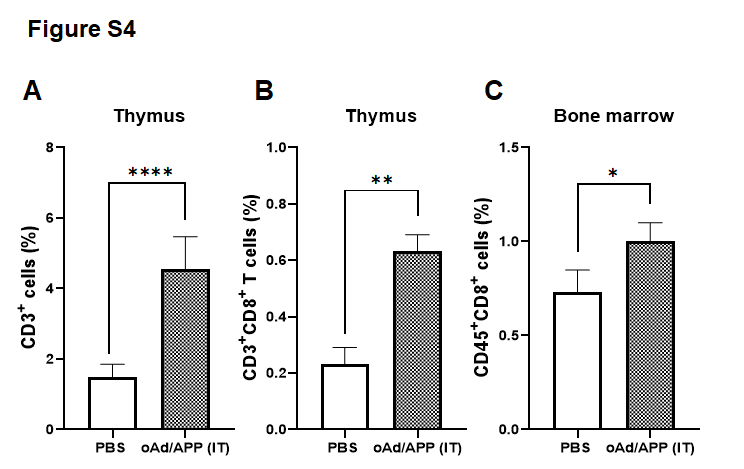

Supplement: Supplementary Figure 4 — Changes in immune cell population within thymus and bone marrow after intratumoral administration of oAd/APP in B16-F10 tumor bearing mice. Thymus (A, B) or bone marrow (C) was harvested at 3 days after the third administration of oAd/APP complex (1 × 10^9^ VP) into B16-F10 tumor-bearing mice CD3 or CD8 expression levels were analyzed by flow cytometry (n =3 mice per group). Each data point indicated mean ± SD. (**P < 0.01, ****P < 0.0001, and non-significant (NS)). [file Image_4.tif]
